# Author Correction: Collagenolysis-dependent DDR1 signalling dictates pancreatic cancer outcome

**DOI:** 10.1038/s41586-025-09285-4

**Published:** 2025-06-18

**Authors:** Hua Su, Fei Yang, Rao Fu, Brittney Trinh, Nina Sun, Junlai Liu, Avi Kumar, Jacopo Baglieri, Jeremy Siruno, Michelle Le, Yuhan Li, Stephen Dozier, Ajay Nair, Aveline Filliol, Nachanok Sinchai, Sara Brin Rosenthal, Jennifer Santini, Christian M. Metallo, Anthony Molina, Robert F. Schwabe, Andrew M. Lowy, David A. Brenner, Beicheng Sun, Michael Karin

**Affiliations:** 1 Laboratory of Gene Regulation and Signal Transduction, Departments of Pharmacology and Pathology, School of Medicine, University of California San Diego, La Jolla, CA USA; 2 Department of Hepatobiliary Surgery, Affiliated Drum Tower Hospital of Nanjing University Medical School, Nanjing, China; 3 Department of Medicine, University of California San Diego, La Jolla, CA USA; 4 Molecular and Cell Biology Laboratory, Salk Institute for Biological Studies, La Jolla, CA USA; 5 Department of Medicine, Columbia University, New York, NY USA; 6 Center for Computational Biology and Bioinformatics, Department of Medicine, University of California San Diego, La Jolla, CA USA; 7 UCSD School of Medicine Microscopy Core, University of California San Diego, La Jolla, CA USA; 8 Department of Surgery, Division of Surgical Oncology, Moores Cancer Center, University of California San Diego, La Jolla, CA USA; 9 Department of Hepatobiliary Surgery, First Affiliated Hospital of Anhui Medical University, Hefei, China

**Keywords:** Cancer metabolism, Cancer microenvironment, Cancer metabolism, Nutrient signalling

Correction to: *Nature* 10.1038/s41586-022-05169-z Published online 5 October 2022

In the version of the article initially published, the middle initial of David A. Brenner was missing and has now been added to the HTML and PDF versions of the article.

