# Supplementary material for: Collagenolysis-dependent DDR1 signalling dictates pancreatic cancer outcome
Source: Nature. 2022 Oct 5;610(7931):366–72. doi: 10.1038/s41586-022-05169-z (PMC9588640; doi:10.1038/s41586-022-05169-z)

nature portfolio

https://doi.org/10.1038/s41586-022-05169-z

Supplementary information

Collagenolysis-dependent DDR1 signalling dictates pancreatic cancer outcome

In the format provided by the authors and unedited

Table 1: CRISPR CAS9 sgRNA Sequence List

Gene	sgRNA sequence (5’-3’)
Ddr1	GTAACGCAACCGATAGCTTC
Mrc2	CCGGTGGACCAATGTCAAGG
Itgb1	AATGTCACCAATCGCAGCAA
Lair1	GTCCGAACGTAGTAAGACGC
Nrf2	GGCATCTTGTTTGGGAATGT
Col1α1	CGTGCAATGCAATGAAGAAC
DDR1	GGATCTACAACGACTGCACC

Table 2: Antibody List

Antibody	Catalogue Number	Company
anti-p62	GP62-C	Progen
anti-NRF2	A11159	ABclonal
anti-COL1A1	72026	CST
	sc-293182	Santa Cruz
anti-3/4 COL1A1	0217-050	Immunoglobe
anti-TIM23	sc-514463	Santa Cruz
Anti-phospho-DDR1 (pTyr513)	SAB4504671	Sigma
anti-DDR1	sc-390268	Santa Cruz
anti-KEAP1	8047	CST
anti-NF-κB p65	8242	CST
anti-Histone H3	A2348	ABclonal
anti-CD326 (EpCAM)	13-5791-80	ThermoFisher
anti-IKKα	MA5-16157	Invitrogen
anti-Actin	A4700	Sigma
anti-GFP	A-11122	ThermoFisher
anti-GFP/YFP/CFP	ab13970	Abcam
anti-Flag	F3165, F7425	Sigma
anti-TFAM	ab131607	Abcam
anti-PGC1α	ABE868	Sigma
anti-Phospho-AMPKα (Thr172)	2535	CST
anti-AMPKα	5832	CST
anti-6X His tag	ab18184	Abcam
anti-E-Cadherin	3195	CST
anti-CD138/SDC1	36-2900	ThermoFisher
anti-NHE-1	sc-136239	Santa Cruz
anti-PI3 Kinase p110γ	5405	CST
anti-ATP5A	sc-136178	Santa Cruz
anti-ATP5B	MAB3494	Sigma
anti-UQCRC2	sc-390378	Santa Cruz
anti-SDHB	sc-271548	Santa Cruz
	92649	CST
anti-NDUFB7	sc-365552	Santa Cruz
anti-COX1/MT-CO1	62101	CST
anti-αSMA	ab5694	Abcam
anti-MMP1	ab52631	Abcam
anti-Ki67	GTX16667	GeneTex
anti-CDC42	PA1-092	ThermoFisher
anti-HSP90	sc-13119	Santa Cruz
anti-α-Amylase	A8273	Sigma
anti-cytokeratin 19	sc-33111	Santa Cruz

Table 2: Antibody List

Antibody	Catalogue Number	Company
anti-SOX9	sc-20095	Santa Cruz
anti-cytokeratin 18	GTX105624	GeneTex
anti-LAIR1	H00003903-D01P	ThermoFisher
anti-Endo180/MRC2	sc-271148	Santa Cruz
anti-Integrin β1/ITGB1	sc-374429	Santa Cruz
anti-CD45	14-0451-85	ThermoFisher
anti-CD68	MA5-13324	ThermoFisher
anti-CD163	ab182422	Abcam
anti-F4/80	MF48000	ThermoFisher
anti-CD4	ab183685	Abcam
anti-Ki67	ab15580	Abcam
anti-CD8	ab217344	Abcam
HRP goat anti-chicken IgY	sc-2428	Santa Cruz
HRP goat anti-rabbit IgG	7074	CST
HRP horse anti-mouse IgG	7076	CST
HRP streptavidin	554066	Pharmingen
Biotin goat anti-mouse IgG	553999	Pharmingen
Biotin goat anti-rabbit IgG	550338	Pharmingen
Biotin mouse anti-goat IgG	sc-2489	Santa Cruz

Table 3: Real-time PCR Primer List

Gene	Forward (5'-3')	Reverse (5'-3')
Pik3ca	GGACTGTGTGGGTCTCATCG	TCTCGCCCTTGTTCTTGTCC
Pik3cg	CTCTGGACCTGTGCCTTCTG	ATCTTTGAATGCCCCCGTGT
Cdc42	GAGACTGCTGAAAAGCTGGCG	GGCTCTTCTTCGGTTCTGGAGG
Nfe2l2	AACAGAACGGCCCTAAAGCA	GGGATTCACGCATAGGAGCA
Nhe1	TCATGAAGATAGGTTTCCATGTGAT	CGTCTGATTGCAGGAAGGGG
Sdc1	TCTGGCTCTGGCTCTGCG	GCCGTGACAAAGTATCTGGC
Sqstm1	TGGGCAAGGAGGAGGCGACC	CCTCATCGCGGTAGTGCGCC
Egf	TTCTCACAAGGAAAGAGCATCTC	GTCCTGTCCCGTTAAGGAAAAC
m18s	AGCCCCTGCCCTTTGTACACA	CGATCCGAGGGCCTCACTA

Supplementary Fig. 1: Raw images of immunoblot and DNA gel

Fig. 1a

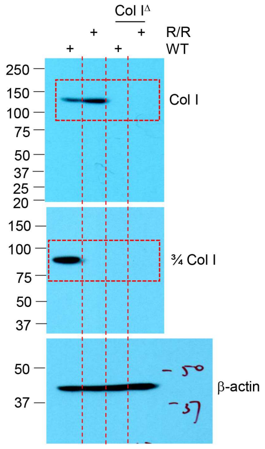

Fig. 3b

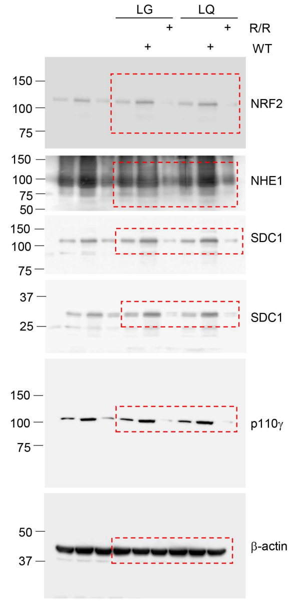

Fig. 4b

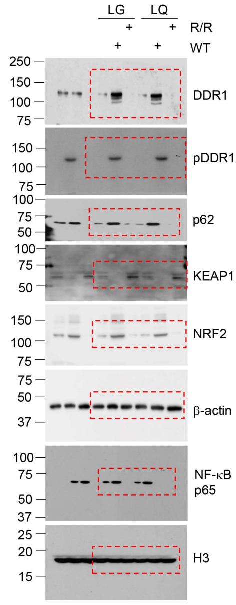

Fig. 4d

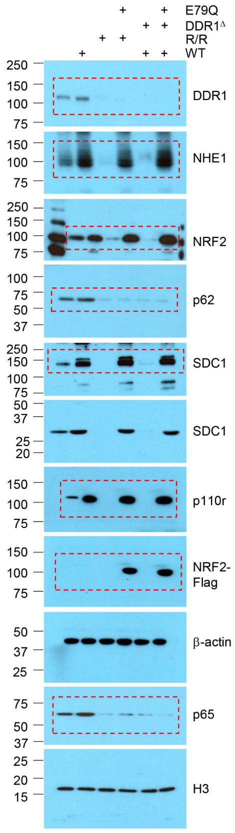

Fig. 4f

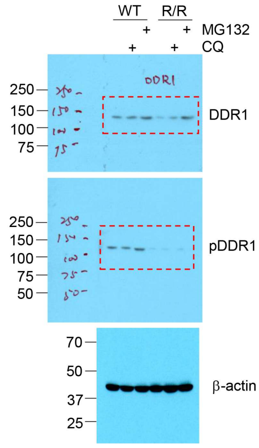

Fig. 6c

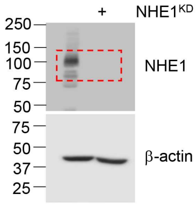

Fig. 6g

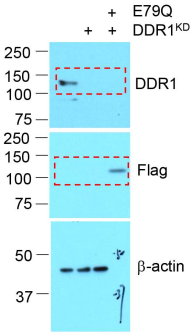

Extended Data Fig. 2b

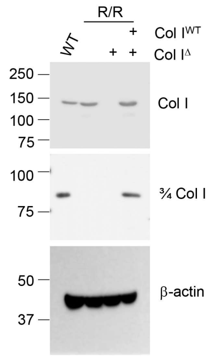

Extended Data Fig. 2c

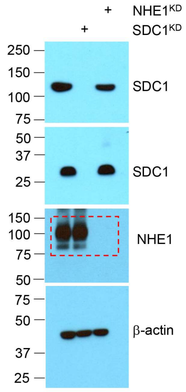

Extended Data Fig. 3f

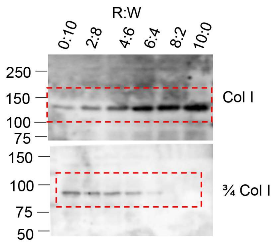

Extended Data Fig. 3g

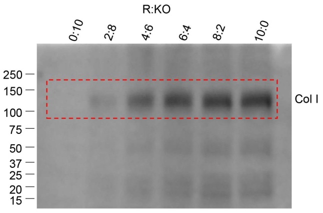

Extended Data Fig. 4a

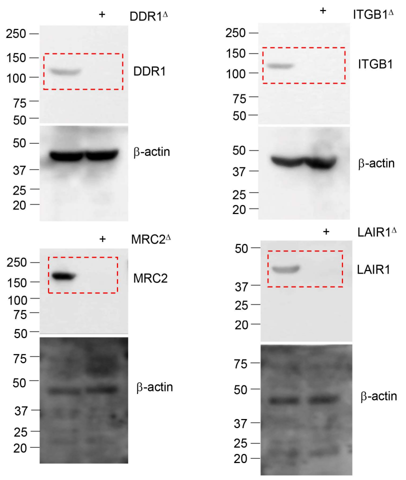

Supplementary Fig. 1: Raw images of immunoblot and DNA gel

Extended Data Fig. 4c

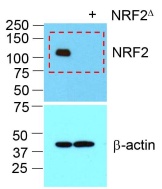

Extended Data Fig. 4f

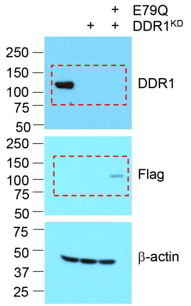

Extended Data Fig. 5a

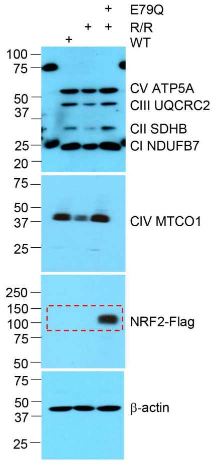

Extended Data Fig. 5b

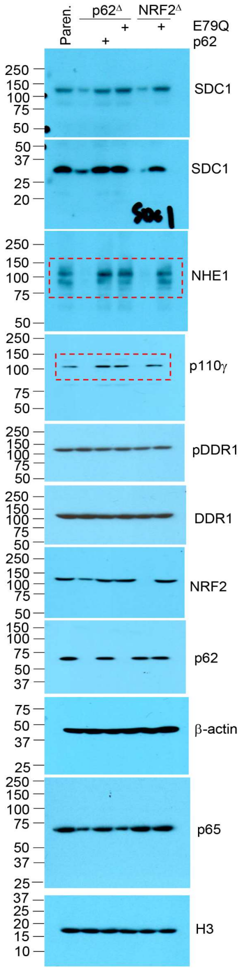

Extended Data Fig. 5c

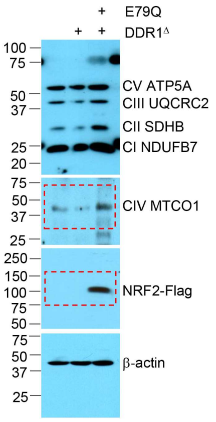

Extended Data Fig. 5d

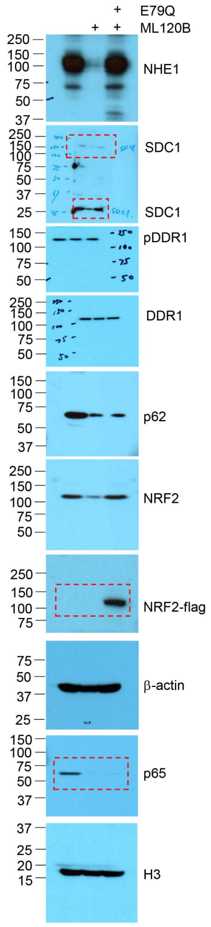

Extended Data Fig. 5e

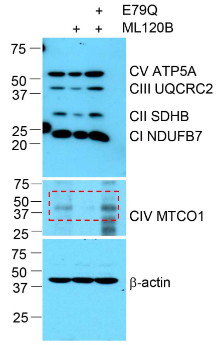

Extended Data Fig. 6b

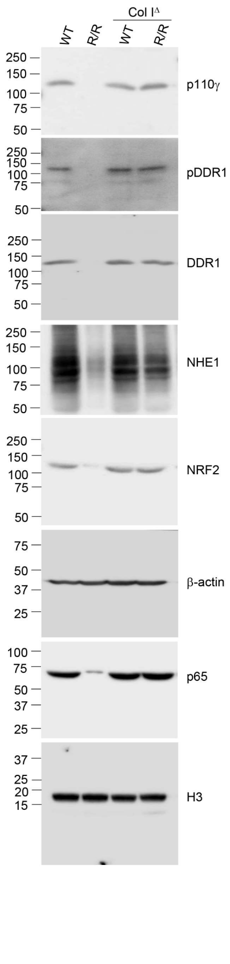

Extended Data Fig. 6f

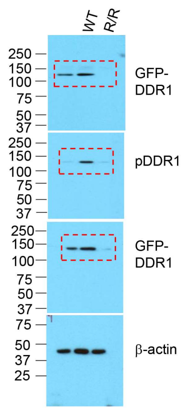

Extended Data Fig. 6k

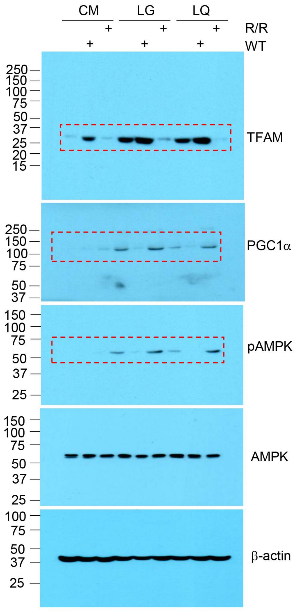

Extended Data Fig. 6d

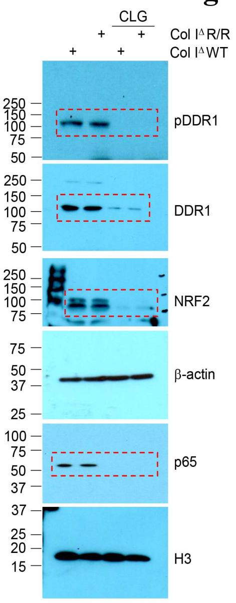

Supplementary Fig. 1: Raw images of immunoblot and DNA gel

Extended Data Fig. 6m (DNA gel)

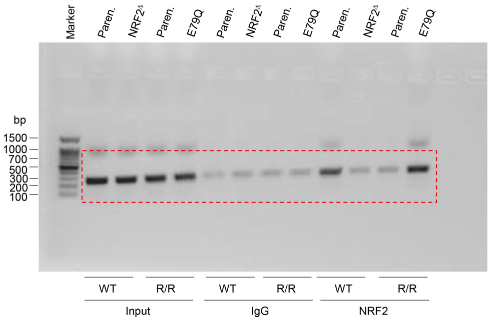

Extended Data Fig. 8b

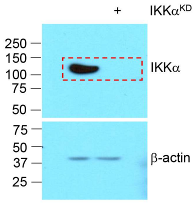

Extended Data Fig. 8i

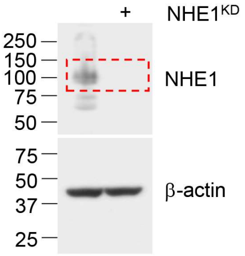

Extended Data Fig. 10c

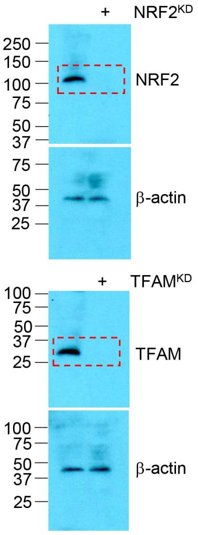

Supplement: Supplementary file 1 — This file contains Supplementary Tables 1–3 and Supplementary Fig. 1 [file 41586_2022_5169_MOESM1_ESM.pdf]
