# Supplementary material for: Collagenolysis-dependent DDR1 signalling dictates pancreatic cancer outcome
Source: Nature. 2022 Oct 5;610(7931):366–72. doi: 10.1038/s41586-022-05169-z (PMC9588640; doi:10.1038/s41586-022-05169-z)
Supplement: Supplementary file 2 — Reporting Summary [file 41586_2022_5169_MOESM2_ESM.pdf]

Corresponding author(s): Michael Karin
Last updated by author(s): Jul 14, 2022

Reporting Summary

Nature Portfolio wishes to improve the reproducibility of the work that we publish. This form provides structure for consistency and transparency in reporting. For further information on Nature Portfolio policies, see our Editorial Policies and the Editorial Policy Checklist.

Statistics

For all statistical analyses, confirm that the following items are present in the figure legend, table legend, main text, or Methods section.

n/a	Confirmed	
☐	☒	The exact sample size (*n*) for each experimental group/condition, given as a discrete number and unit of measurement
☐	☒	A statement on whether measurements were taken from distinct samples or whether the same sample was measured repeatedly
☐	☒	The statistical test(s) used AND whether they are one- or two-sided<br>*Only common tests should be described solely by name; describe more complex techniques in the Methods section.*
☒	☐	A description of all covariates tested
☒	☐	A description of any assumptions or corrections, such as tests of normality and adjustment for multiple comparisons
☐	☒	A full description of the statistical parameters including central tendency (e.g. means) or other basic estimates (e.g. regression coefficient) AND variation (e.g. standard deviation) or associated estimates of uncertainty (e.g. confidence intervals)
☐	☒	For null hypothesis testing, the test statistic (e.g. *F*, *t*, *r*) with confidence intervals, effect sizes, degrees of freedom and *P* value noted<br>*Give P values as exact values whenever suitable.*
☒	☐	For Bayesian analysis, information on the choice of priors and Markov chain Monte Carlo settings
☒	☐	For hierarchical and complex designs, identification of the appropriate level for tests and full reporting of outcomes
☒	☐	Estimates of effect sizes (e.g. Cohen's *d*, Pearson's *r*), indicating how they were calculated

Our web collection on statistics for biologists contains articles on many of the points above.

Software and code

Policy information about availability of computer code

Data collection	SoftMax 6.5, Leica Application Suite AF 2.6.0.7266, GEPIA2, AxioVision Rel. 4.5
Data analysis	SoftMax 6.5, Leica Application Suite AF 2.6.0.7266, Graphpad Prism 9, GEPIA2, Image J 1.53k, Fiji Image J 1.53q, R (v4.0.2), Seurat4 (v4.0.5), CT-Fire software (v.2.0 beta), HOMER (v4.11), GSEA (4.0.3)

For manuscripts utilizing custom algorithms or software that are central to the research but not yet described in published literature, software must be made available to editors and reviewers. We strongly encourage code deposition in a community repository (e.g. GitHub). See the Nature Portfolio guidelines for submitting code & software for further information.

Data

Policy information about availability of data

All manuscripts must include a data availability statement. This statement should provide the following information, where applicable:

- Accession codes, unique identifiers, or web links for publicly available datasets
- A description of any restrictions on data availability
- For clinical datasets or third party data, please ensure that the statement adheres to our policy

RNA-seq data for KPC960 cells grown on WT or R/R ECM have been deposited in the Gene Expression Omnibus under the accession code GSE206218, scRNA-seq dataset for 5 primary PDAC tumors and 1 PDAC liver metastasis were obtained from the published GEO dataset (GSE156405) (Lee et al., Clinical Cancer Research, 2021).Custom computer code used in above scRNA-seq analysis is available at https://github.com/ajynair/Collagen_DDR1_PDACmets.The mouse reference genome

GRCm38 was obtained from Ensembl version 86 or 102 (http://nov2020.archive.ensembl.org/Mus_musculus/Info/Index). TCGA data are available within GEPIA2 (http://gepia2.cancer-pku.cn/#dataset).

Human research participants

Policy information about studies involving human research participants and Sex and Gender in Research.

Reporting on sex and gender

no sex- and gender-based analyses have been performed. This study did not involve sex or gender research.

Population characteristics

Human PDAC specimens were acquired from patients who were diagnosed with PDAC between January 2017 and May 2021 at The Affiliated Drum Tower Hospital of Nanjing University Medical School (Nanjing, Jiangsu, China). All patients received standard surgical resection and did not receive chemotherapy before surgery. Paraffin embedded tissues were processed by a pathologist after surgical resection and confirmed as PDAC prior to further investigation. Overall survival duration was defined as the time from date of diagnosis to that of death or last known follow-up examination. Survival information was available for 81 of the 106 patients. Patient number and age in the parentheses are listed as follows,
#21-41(55)
#21-42(57)
#21-43(58)
#21-44(52)
#21-45(52)
#21-46(54)
#21-47(57)
#21-48(58)
#21-49(53)
#21-50(68)
#21-51(40)
#21-52(57)
#21-53(51)
#21-54(53)
#21-55(55)
#21-56(63)
#21-57(64)
#21-58(57)
#21-59(55)
#21-60(58)
#19-11(69)
#19-12(65)
#19-14(62)
#19-16(68)
#19-20(61)
#19-21(73)
#19-22(66)
#19-23(71)
#19-24(63)
#19-25(74)
#19-26(72)
#19-27(74)
#19-28(52)
#19-29(56)
#19-30(60)
#19-31(53)
#19-32(62)
#19-33(70)
#19-34(54)
#19-35(68)
#19-37(49)
#19-39(81)
#19-40(67)
#19-43(53)
#19-44(57)
#19-45(76)
#19-46(62)
#19-47(57)
#19-48(71)
#19-49(70)
#19-50(49)
#19-52(57)
#19-54(70)
#19-55(73)
#19-56(65)
#19-57(77)
#19-58(62)

#19-60(49)
#19-61(77)
#19-62(41)
#19-63(69)
#19-64(62)
#19-65(64)
#19-66(63)
#19-67(71)
#19-68(64)
#19-69(67)
#19-70(67)
#19-71(61)
#19-72(66)
#19-73(61)
#19-74(64)
#19-75(73)
#19-76(66)
#19-77(58)
#19-78(67)
#19-79(65)
#19-80(76)
#19-81(81)
#19-82(57)
#19-83(64)
#19-84(50)
#19-85(50)
#19-86(54)
#19-87(75)
#19-88(82)
#19-89(55)
#19-90(71)
#19-91(61)
#19-92(75)
#19-93(64)
#19-95(48)
#19-97(49)
#19-100(67)
#19-101(61)
#19-103(78)
#19-104(85)
#19-105(55)
#19-107(69)
#19-108(87)
#19-109(67)
#19-110(75)
#19-111(58)
#19-112(44)
#19-113(58)
#19-115(57)
#19-116(57)

Recruitment

Human PDAC specimens were acquired from patients who were diagnosed with PDAC between January 2017 and May 2021 at The Affiliated Drum Tower Hospital of Nanjing University Medical School (Nanjing, Jiangsu, China). All patients received standard surgical resection and did not receive chemotherapy before surgery. Paraffin embedded tissues were processed by a pathologist after surgical resection and confirmed as PDAC prior to further investigation. Informed consent for tissue analysis was obtained before surgery.

Ethics oversight

The study was approved by the Institutional Ethics Committee of The Affiliated Drum Tower Hospital with IRB #2021-608-01.

Note that full information on the approval of the study protocol must also be provided in the manuscript.

Field-specific reporting

Please select the one below that is the best fit for your research. If you are not sure, read the appropriate sections before making your selection.

☒ Life sciences ☐ Behavioural & social sciences ☐ Ecological, evolutionary & environmental sciences

For a reference copy of the document with all sections, see nature.com/documents/nr-reporting-summary-flat.pdf

Life sciences study design

All studies must disclose on these points even when the disclosure is negative.

Sample size	No statistical methods were used to predetermine samples sizes for in vitro experiments. Sample sizes were chosen in order to be able to perform statistical analyses, as is standard in the field and based on previous studies (Su et al., Cancer Cell, 2021; Zhong et al., Nature, 2018). For in vivo experiments, based on their genotypes, gender- and age matched mice were randomly allocated to experimental groups. Because our mice were inbred and age- and gender-matched, similar variance was assumed between different experimental groups. No sample size pre-estimation was performed but we used as many mice per group as possible to minimize type I/II errors.
Data exclusions	No data were excluded for all the analyses described.
Replication	All the experiments except IHC analysis of 106 patient samples were repeated for at least three times. However, all the antibodies used in IHC analysis of 106 patient samples were confirmed their specificity in several patient samples at least three times. Statistical analysis were done to ensure significance. All attempts of replication were successful.
Randomization	Age- , gender-, and equal average tumor volumes-matched mice were randomly allocated to different experimental groups based on their genotypes. For experiments other than mice, we did not carry out randomization because it's either irrelevant or not applicable to these studies.
Blinding	Investigators were not blinded to the group allocations except for microscopic analysis of immunofluorescent or IHC staining results. For other experiments, the investigators were not blinded since analyses relied on unbiased measurements of quantitative parameters. Standardized procedures for data collection and analysis were used to prevent bias.

Reporting for specific materials, systems and methods

We require information from authors about some types of materials, experimental systems and methods used in many studies. Here, indicate whether each material, system or method listed is relevant to your study. If you are not sure if a list item applies to your research, read the appropriate section before selecting a response.

Materials & experimental systems

n/a	Involved in the study	
☐	☒	Antibodies
☐	☒	Eukaryotic cell lines
☒	☐	Palaeontology and archaeology
☐	☒	Animals and other organisms
☒	☐	Clinical data
☒	☐	Dual use research of concern

Methods

n/a	Involved in the study	
☒	☐	ChIP-seq
☒	☐	Flow cytometry
☒	☐	MRI-based neuroimaging

Antibodies

Antibodies used	Guinea pig anti-p62 polyclonal antibody (GP62-C, Progen), rabbit anti-NRF2 polyclonal antibody (ABclonal, A11159), rabbit anti-COL1A1 monoclonal antibody (CST, 72026, E8F4L), mouse anti-COL1A1 monoclonal antibody (Santa Cruz, sc-293182, 3G3), rabbit anti-3/4 COL1A1 polyclonal antibody (Immunoglobe, 0217-050), mouse anti-TIM23 monoclonal antibody (Santa Cruz, sc-514463, H-8), rabbit anti-phospho-DDR1 (pTyr513) polyclonal antibody (Sigma, SAB4504671), mouse anti-DDR1 monoclonal antibody (Santa Cruz, sc-390268, D-10), rabbit anti-KEAP1 monoclonal antibody (CST, 8047, D6B12), rabbit anti-NF-κB p65 monoclonal antibody (CST, 8242, D14E12), rabbit anti-Histone H3 polyclonal antibody (ABclonal, A2348), rat anti-CD326 (EpCAM) monoclonal antibody (ThermoFisher, 13-5791-80, G8.8), mouse anti-IKKa monoclonal antibody (Invitrogen, MA5-16157, 14A231), mouse anti-Actin monoclonal antibody (Sigma, A4700, AC-40), rabbit anti-GFP polyclonal antibody (ThermoFisher, A-11122), chicken anti-GFP/YFP/CFP polyclonal antibody (Abcam ab13970), mouse anti-Flag monoclonal antibody (Sigma, F3165, M2), rabbit anti-Flag polyclonal antibody (Sigma, F7425), rabbit anti-TFAM polyclonal antibody (Abcam, ab131607), rabbit anti-PGC1 polyclonal antibody (Sigma, ABE868), rabbit anti-Phospho-AMPKα (Thr172) monoclonal antibody (CST, 2535, 40H9), rabbit anti-AMPKα monoclonal antibody (CST, 5832, D63G4), mouse anti-6X His tag monoclonal antibody (Abcam, ab18184, HIS.H8), rabbit anti-E-Cadherin monoclonal antibody (CST, 3195, 24E10), rabbit anti-CD138/SDC1 polyclonal antibody (ThermoFisher, 36-2900), mouse anti-NHE-1 monoclonal antibody (Santa Cruz, sc-136239, 54), rabbit anti-PI3 Kinase p110γ monoclonal antibody (CST, 5405, D55D5), mouse anti-ATP5A monoclonal antibody (Santa Cruz, sc-136178, 51), mouse anti-ATP5B monoclonal antibody (Sigma, MAB3494, 4.3E8.D1), mouse anti-UQCRC2 monoclonal antibody (Santa Cruz, sc-390378, G-10), mouse anti-SDHB monoclonal antibody (Santa Cruz, sc-271548, G-10), rabbit anti-SDHB monoclonal antibody (CST, 92649, E3H9Z), mouse anti-NDUFB7 monoclonal antibody (Santa Cruz, sc-365552, F-8), rabbit anti-COX1/MT-CO1 polyclonal antibody (CST, 62101), rabbit anti- SMA polyclonal antibody (Abcam, ab5694), rabbit anti-MMP1 monoclonal antibody (Abcam, ab52631, EP1247Y), rabbit anti-Ki67 monoclonal antibody (GeneTex, GTX16667, SP6), rabbit anti-CDC42 polyclonal antibody (ThermoFisher, PA1-092), mouse anti-HSP90 monoclonal antibody (Santa Cruz, sc-13119, F-8), rabbit anti-α-Amylase polyclonal antibody (Sigma, A8273), goat anti-cytokeratin 19 polyclonal antibody (Santa Cruz, sc-33111), rabbit anti-SOX9 polyclonal antibody (Santa Cruz, sc-20095), mouse anti-cytokeratin 18 polyclonal antibody (GeneTex, GTX105624), rabbit anti-LAIR1 polyclonal antibody (ThermoFisher, H00003903-D01P), mouse anti-Endo180/MRC2 monoclonal antibody (Santa Cruz, sc-271148, B-10), mouse anti-Integrin β1/ITGB1 monoclonal antibody (Santa Cruz, sc-374429, A-4), Rat anti-CD45 monoclonal antibody (ThermoFisher, 14-0451-85, 30-F11), Mouse anti-CD68 monoclonal antibody (ThermoFisher, MA5-13324, KP1), Rabbit anti-CD163 monoclonal

antibody (Abcam, ab182422, EPR19518), Rat anti-F4/80 monoclonal antibody (ThermoFisher, MF48000, BM8), Rabbit anti-CD4 monoclonal antibody (Abcam, ab183685, EPR19514), Rabbit anti-Ki67 polyclonal antibody (Abcam, ab15580), Rabbit anti-CD8 monoclonal antibody (Abcam, ab217344, EPR21769), HRP goat anti-chicken IgY antibody (Santa Cruz, sc-2428), HRP goat anti-rabbit IgG antibody (CST, 7074), HRP horse anti-mouse IgG antibody (CST, 7076), HRP streptavidin (Pharmingen, 554066), Biotin goat anti-mouse IgG (Pharmingen, 553999), Biotin goat anti-rabbit IgG (Pharmingen, 550338), Biotin mouse anti-goat IgG (Santa Cruz, sc-2489). Alexa 594-, Alexa 647-, and Alexa 488-conjugated secondary antibodies were used: donkey anti-mouse IgG, donkey anti-rabbit IgG, goat anti-chicken IgY (Molecular Probes, Invitrogen).

Validation

All the following antibodies have been validated according to manufacturer's manuals and re-validated by immunoblot (IB), or immunofluorescence staining (IF) or immunohistochemistry (IHC) results from this manuscript:
Guinea pig anti-p62 polyclonal antibody (GP62-C, Progen) (IB, human and mouse): https://us.progen.com/anti-p62-SQSTM1-C-terminus-guinea-pig-polyclonal-serum/GP62-C
rabbit anti-NRF2 polyclonal antibody (ABclonal, A11159) (IB, IHC, IF, human and mouse): https://abclonal.com/catalog-antibodies/NRF2RabbitpAb/A11159
rabbit anti-COL1A1 monoclonal antibody (CST, 72026) (IB, human and mouse): https://www.cellsignal.com/products/primary-antibodies/col1a1-e8f4l-xp-rabbit-mab/72026
mouse anti-COL1A1 monoclonal antibody (Santa Cruz, sc-293182) (IB, human and mouse): https://www.scbt.com/p/col1a1-antibody-3g3
rabbit anti-3/4 COL1A1 polyclonal antibody (Immunoglobe, 0217-050) (IB, IF, IHC, human and mouse): https://www.immunoglobe.com/antibodies/items/collagen_cleavage_site.html
mouse anti-TIM23 monoclonal antibody (Santa Cruz, sc-514463) (IF, human and mouse): https://www.scbt.com/p/tim23-antibody-h-8
rabbit anti-phospho-DDR1 (pTyr513) polyclonal antibody (Sigma, SAB4504671) (IB, human and mouse): https://www.sigmaaldrich.com/US/en/product/sigma/sab4504671
mouse anti-DDR1 monoclonal antibody (Santa Cruz, sc-390268) (IB, human and mouse): https://www.scbt.com/p/ddr1-antibody-d-10
rabbit anti-KEAP1 monoclonal antibody (CST, 8047) (IB, human and mouse): https://www.cellsignal.com/products/primary-antibodies/keap1-d6b12-rabbit-mab/8047
rabbit anti-NF-κB p65 monoclonal antibody (CST, 8242) (IB, human and mouse): https://www.cellsignal.com/products/primary-antibodies/nf-kb-p65-d14e12-xp-rabbit-mab/8242
rabbit anti-Histone H3 polyclonal antibody (ABclonal, A2348) (IB, human and mouse): https://abclonal.com/catalog-antibodies/HistoneH3RabbitpAb/A2348
rat anti-CD326 (EpCAM) monoclonal antibody (ThermoFisher, 13-5791-80) (IF, IHC, mouse): https://www.thermofisher.com/antibody/product/CD326-EpCAM-Antibody-clone-G8-8-Monoclonal/13-5791-80
mouse anti-IKKa monoclonal antibody (Invitrogen, MA5-16157) (IB, human and mouse): https://www.thermofisher.com/antibody/product/IKK-alpha-Antibody-clone-14A231-Monoclonal/MA5-16157
mouse anti-Actin monoclonal antibody (Sigma, A4700) (IB, human and mouse): https://www.sigmaaldrich.com/US/en/product/sigma/a4700
rabbit anti-GFP polyclonal antibody (ThermoFisher, A-11122) (IB):https://www.thermofisher.com/antibody/product/GFP-Antibody-Polyclonal/A-11122
chicken anti-GFP/YFP/CFP polyclonal antibody (Abcam ab13970) (IF): https://www.abcam.com/gfp-antibody-ab13970.html
mouse anti-Flag monoclonal antibody (Sigma, F3165) (IB, human and mouse): https://www.sigmaaldrich.com/US/en/product/sigma/f3165
rabbit anti-Flag polyclonal antibody (Sigma, F7425) (IB, human and mouse): https://www.sigmaaldrich.com/US/en/product/sigma/f7425
rabbit anti-TFAM polyclonal antibody (Abcam, ab131607) (IB, mouse): https://www.citeab.com/antibodies/754337-ab131607-anti-mttfa-antibody-mitochondrial-marker
rabbit anti-PGC1a polyclonal antibody (Sigma, ABE868) (IB, human and mouse): https://www.sigmaaldrich.com/US/en/product/mm/abe868
rabbit anti-Phospho-AMPKα (Thr172) monoclonal antibody (CST, 2535) (IB, human and mouse): https://www.cellsignal.com/products/primary-antibodies/phospho-ampka-thr172-40h9-rabbit-mab/2535
rabbit anti-AMPKα monoclonal antibody (CST, 5832) (IB, human and mouse): https://www.cellsignal.com/products/primary-antibodies/ampka-d63g4-rabbit-mab/5832
mouse anti-6X His tag monoclonal antibody (Abcam, ab18184) (IB, mouse): https://www.abcam.com/6x-his-tag-antibody-hish8-ab18184.html
rabbit anti-E-Cadherin monoclonal antibody (CST, 3195) (IF, human and mouse): https://www.cellsignal.com/products/primary-antibodies/e-cadherin-24e10-rabbit-mab/3195
rabbit anti-CD138/SDC1 antibody (ThermoFisher, 36-2900) (IB, human and mouse): https://www.thermofisher.com/antibody/product/CD138-Antibody-Polyclonal/36-2900
mouse anti-NHE-1 monoclonal antibody (Santa Cruz, sc-136239) (IB, human and mouse): https://www.scbt.com/p/nhe-1-antibody-54
rabbit anti-PI3 Kinase p110γ monoclonal antibody (CST, 5405) (IB, human and mouse): https://www.cellsignal.com/products/primary-antibodies/pi3-kinase-p110g-d55d5-rabbit-mab/5405
mouse anti-ATP5A monoclonal antibody (Santa Cruz, sc-136178) (IB, human and mouse): https://www.scbt.com/p/atp5a-antibody-51
mouse anti-ATP5B monoclonal antibody (Sigma, MAB3494) (IB, human and mouse): https://www.sigmaaldrich.com/deepweb/assets/sigmaaldrich/product/documents/309/124/mab3494.pdf
mouse anti-UQCRC2 monoclonal antibody (Santa Cruz, sc-390378) (IB, human and mouse): https://www.scbt.com/p/uqcrc2-antibody-g-10
mouse anti-SDHB monoclonal antibody (Santa Cruz, sc-271548) (IB, human and mouse): https://www.scbt.com/p/sdhb-antibody-g-10
rabbit anti-SDHB monoclonal antibody (CST, 92649) (IHC, human and mouse): https://www.cellsignal.com/products/primary-antibodies/sdhb-e3h9z-xp-rabbit-mab/92649
mouse anti-NDUFB7 monoclonal antibody (Santa Cruz, sc-365552) (IB, human and mouse): https://www.scbt.com/p/ndufb7-antibody-f-8
rabbit anti-COX1/MT-CO1 polyclonal antibody (CST, 62101) (IB, human and mouse): https://www.cellsignal.com/products/primary-antibodies/cox1-mt-co1-antibody/62101

rabbit anti-aSMA polyclonal antibody (Abcam, ab5694) (IHC, mouse and human): https://www.abcam.com/alpha-smooth-muscle-actin-antibody-ab5694.html
rabbit anti-MMP1 monoclonal antibody (Abcam, ab52631) (IHC, human): https://www.abcam.com/mmp1-antibody-ep1247y-ab52631.html
rabbit anti-Ki67 monoclonal antibody (GeneTex, GTX16667) (IHC, human and mouse): https://www.genetex.com/Product/Detail/Ki67-antibody-SP6/GTX16667
rabbit anti-CDC42 polyclonal antibody (ThermoFisher, PA1-092) (IHC, IB, human and mouse): https://www.thermofisher.com/antibody/product/Cdc42-Antibody-Polyclonal/PA1-092
mouse anti-HSP90 monoclonal antibody (Santa Cruz, sc-13119) (IB, human and mouse): https://www.scbt.com/p/hsp-90alpha-beta-antibody-f-8
rabbit anti-α-Amylase polyclonal antibody (Sigma, A8273) (IHC, human): https://www.sigmaaldrich.com/US/en/product/sigma/a8273
mouse anti-cytokeratin 18 monoclonal antibody (GeneTex, GTX105624) (IF, human and mouse): https://www.genetex.com/Product/Detail/Cytokeratin-18-antibody-N2C2-Internal/GTX105624
rabbit anti-LAIR1 polyclonal antibody (ThermoFisher, H00003903-D01P) (IB, human and mouse): https://www.thermofisher.com/antibody/product/LAIR1-Antibody-Polyclonal/H00003903-D01P
mouse anti-Endo180/MRC2 monoclonal antibody (Santa Cruz, sc-271148) (IB, human): https://www.scbt.com/p/endo180-antibody-b-10
mouse anti-Integrin β1/ITGB1 antibody (Santa Cruz, sc-374429) (IB, human and mouse): https://www.scbt.com/p/integrin-beta1-antibody-a-4
Rat anti-CD45 antibody (ThermoFisher, 14-0451-85) (IHC, mouse): https://www.thermofisher.com/antibody/product/CD45-Antibody-clone-30-F11-Monoclonal/14-0451-85
Mouse anti-CD68 antibody (ThermoFisher, MA5-13324) (IHC, human): https://www.thermofisher.com/antibody/product/CD68-Antibody-clone-KP1-Monoclonal/MA5-13324
Rabbit anti-CD163 antibody (Abcam, ab182422) (IHC, human and mouse): https://www.abcam.com/cd163-antibody-epr19518-ab182422.html
Rat anti-F4/80 antibody (ThermoFisher, MF48000) (IHC, mouse): https://www.thermofisher.com/antibody/product/F4-80-Antibody-clone-BM8-Monoclonal/MF48000
Rabbit anti-CD4 antibody (Abcam, ab183685) (IHC, mouse): https://www.abcam.com/cd4-antibody-epr19514-ab183685.html
Rabbit anti-Ki67 antibody (Abcam, ab15580) (IHC, mouse and human): https://www.abcam.com/ki67-antibody-ab15580.html
Rabbit anti-CD8 antibody (Abcam, ab217344) (IHC, mouse): https://www.abcam.com/cd8-alpha-antibody-epr21769-ab217344.html
HRP goat anti-chicken IgY antibody (Santa Cruz, sc-2428) (IF): https://datasheets.scbt.com/sc-2428.pdf
HRP goat anti-rabbit IgG antibody (CST, 7074) (IB): https://www.cellsignal.com/products/secondary-antibodies/anti-rabbit-igg-hrp-linked-antibody/7074
HRP horse anti-mouse IgG antibody (CST, 7076) (IB): https://www.cellsignal.com/products/secondary-antibodies/anti-mouse-igg-hrp-linked-antibody/7076
HRP streptavidin (Pharmingen, 554066) (IHC): https://www.bdbiosciences.com/content/dam/bdb/products/global/reagents/immunoassay-reagents/elisa/554066_base/pdf/554066.pdf
Biotin goat anti-mouse IgG (Pharmingen, 553999) (IHC, mouse): https://www.bdbiosciences.com/content/dam/bdb/products/global/reagents/flow-cytometry-reagents/research-reagents/single-color-antibodies-ruo/553999_base/pdf/553999.pdf
Biotin goat anti-rabbit IgG (Pharmingen, 550338) (IHC): https://www.bdbiosciences.com/content/dam/bdb/products/global/reagents/flow-cytometry-reagents/research-reagents/single-color-antibodies-ruo/550338_base/pdf/550338.pdf
Biotin mouse anti-goat IgG (Santa Cruz, sc-2489) (IHC): https://www.scbt.com/p/mouse-anti-goat-igg-b
Alexa 594-, Alexa 647-, and Alexa 488-conjugated secondary antibodies were used: donkey anti-mouse IgG, donkey anti-rabbit IgG, goat anti-chicken IgY (Molecular Probes, Invitrogen) (IF): https://www.thermofisher.com/us/en/home/life-science/antibodies/secondary-antibodies/fluorescent-secondary-antibodies/alexa-fluor-plus-secondary-antibodies.html

Eukaryotic cell lines

Policy information about cell lines and Sex and Gender in Research

Cell line source(s)	MIA PaCa-2 (CRL-1420; RRID: CVCL_0428) cell was obtained from ATCC. UN-KC-6141 (RRID: CVCL_1U11) and UN-KPC-960 (RRID: CVCL_1U12) were obtained from Surinder K. Batra. WT and R/R fibroblasts were generated at Dr. David Brenner lab. 1305 primary human PDAC cells were generated by Dr. Andrew M. Lowy lab from a human PDAC PDX.
Authentication	MIA Paca-2 has been authenticated by ATCC and UN-KC-6141 and UN-KPC-960 have been authenticated by Surinder K. Batra lab before delivery to our lab. And cell lines are routinely authenticated in-house by cell morphology.
Mycoplasma contamination	All cell lines are routinely tested negative for mycoplasma contamination.
Commonly misidentified lines (See ICLAC register)	No commonly misidentified cell lines were used.

Animals and other research organisms

Policy information about studies involving animals; ARRIVE guidelines recommended for reporting animal research, and Sex and Gender in Research

Laboratory animals	Female homozygous Nu/Nu nude mice and C57BL/6 mice were obtained at 6 weeks of age from Charles River Laboratories and The Jackson Laboratory, respectively. 3-month-old Col1a1+/+ (Col IWT) or Col1a1r/r (Col Ir/r) mice on a C57BL/6 background obtained from Dr. David Brenner at UCSD were used in this study (indicated in the Methods) and and were previously described. Age- and sex-matched (except where otherwise indicated) male and female mice of each genotype were generated as littermates for use in

experiments in which different genotypes were compared. All mice were maintained in filter-topped cages on autoclaved food and water, and experiments were performed in accordance with UCSD Institutional Animal Care and Use Committee and NIH guidelines and regulations on age and gender-matched littermates. Dr. Karin's Animal Protocol S00218 was approved by the UCSD Institutional Animal Care and Use Committee. Mice were housed in well filter-topped cages in constant temperature, humidity and pathogen-free controlled environment (23°C ± 2°C, 50-60%), with a standard 12 h light/ 12 h dark cycle, plenty of water and food in their cages, which were described in Methods section.

Wild animals	The study did not involve wild animals.
Reporting on sex	This study did not involve sex research. But Sex-matched male and female mice of each genotype were generated as littermates for use in experiments in which different genotypes were compared.
Field-collected samples	The study did not involve field-collected samples.
Ethics oversight	Dr. Karin's Animal Protocol S00218 was approved by the UCSD Institutional Animal Care and Use Committee

Note that full information on the approval of the study protocol must also be provided in the manuscript.
